# Attacking Profiles of the Best Ranked Teams From Elite Futsal Leagues

**DOI:** 10.3389/fpsyg.2019.01370

**Published:** 2019-06-20

**Authors:** César Méndez, Bruno Gonçalves, Joao Santos, J. N. Ribeiro, Bruno Travassos

**Affiliations:** ^1^Physical Activity and Sport Sciences, Technical University of Madrid, Madrid, Spain; ^2^CIDESD, Research Center in Sports Sciences, Health Sciences and Human Development, Department of Sport Sciences, University of Trás-os Montes e Alto Douro, Vila Real, Portugal; ^3^CIDESD, Research Center in Sports Sciences, Health Sciences and Human Development, Department of Sport Sciences, University of Beira Interior, Covilhã, Portugal

**Keywords:** performance analysis, task-related variables, situational variables, match outcome, discriminant analysis

## Abstract

This study aimed to (i) explore the discriminatory power of the task-related variables and the context in establishing differences in the elite futsal leagues of Portugal, Spain, and Russia and (ii) understand how these variables vary according to the match outcome. Methodological issues concerning efficiency (goals and shots), offensive organisation (positional attack, counterattack, set pieces, or 5vs4+Goalkeeper), 1st goal scored during matches (home or away team), match type (balanced or unbalanced), and match outcome (winner, loser, or drawer) were discussed. Archival data were obtained from the 2017–2018 season of Portuguese, Spanish, and Russian professional futsal leagues for all play-off matches. Crosstabs analysis was conducted to establish the significance relationship between the elite futsal leagues and the situational variables. Afterward, discriminant analysis was used to identify the task-related variables that maximise mean differences between different league teams for defining offensive profile, and the variations found when the condition of the winner, loser, or drawer is taken into account. The results allowed to understand that the Portuguese and Russian teams used the positional attacks more, and less the counterattacks and set pieces than the Spaniards, who present a more balanced offensive profile. Overall, winners were better discriminated by goals scored, whereas 5vs4+Goalkeeper strategy discriminated loser teams. Coaches should be aware of these different offensive profiles in order to increase control over the match planning and decrease predictability against opposing teams.

## Introduction

Futsal is a team sport that involves quick actions and precise movements according to the required physical, technical, and tactical game demands (Castagna et al., 2009). As in other team sports, the constant variability in the contexts of performance in short spaces requires great tactical expertise on the part of the players (Amaral and Garganta, 2005). That is, players should constantly explore the game possibilities for action forged by the spatial–temporal relations with teammates and opponents in relation to the strategic plans defined by coaches, the context of the game, or even the game result (Corrêa et al., 2012; Travassos et al., 2016).

Besides game complexity, in order to identify some regularities that characterise game demands and dynamics, previous research from a performance analysis perspective revealed that there is a need to understand the influence of different types of attacking play (i.e., counterattack, positional attack, the set pieces, etc.) on team effectiveness (Lima-Duarte, 2008; Fukuda and Santana, 2012; Lapresa et al., 2013; Gómez et al., 2015; Sarmento et al., 2016). In line with that, previous research revealed that counterattack, positional attack, set pieces, and 5vs4+Goalkeeper (GK), in this order, are the most successful types of attacking play in futsal (Silva et al., 2004; Botelho and Coppi, 2010; Marchi et al., 2010; Fukuda and Santana, 2012; Poffo and Lima, 2012; Soares-Leite, 2012). Also, when ball possession effectiveness of 4vs4 and 5vs4+GK was analysed without considering the types of attacking play used, the 5vs4+GK revealed higher effectiveness than the 4vs4 (Vicente-Vila and Lago-Peñas, 2016). However, recent research showed that the use of the 5vs4+GK game strategy does not cause changes in the match status, and the teams that were losing and used the 5vs4+GK game strategy lost the game in 93% of cases and received more goals compared to those scored (Méndez, 2017). Regarding the number of shots obtained, without considering efficacy, it was observed that positional attack revealed higher values of shots than the counterattacks and set pieces (Orta et al., 2000; Soares-Leite, 2012; Coelho et al., 2013). The discrepancies observed could be related to the sample size, the level of the teams analysed, or even with the percentage of data in relation to the total number of goals or shots considering all possible types of offensive organisation.

In spite of previous results, team sports performance and effectiveness cannot be viewed in a general way but need to be contextualised to game dynamics according to the specific context of performance (Gómez et al., 2013). In this perspective, research has pointed to the importance of situational variables in team performance (Gómez et al., 2013). For instance, the first goal scored in the match (i.e., home or away team having scored before) is clearly linked to the match outcome (i.e., winner, loser, or drawer) (Armatas et al., 2009; Tenga, 2013). The scoring of the first goal in an elite futsal match determines the winner status of the game in 60–70% of the games evaluated (Sampedro and Prieto, 2012; Soares-Leite, 2013; Soares-Leite, 2014). Also, the match type (i.e., balanced or unbalanced matches were determined by the points or goals difference in the scoreboard. It was established based on previous research according to the opinion of expert coaches. That is, the match outcome exceeding the 10-point differences in basketball and two goals in futsal were considered unbalanced matches) has been investigated as a discriminating factor of the main performance indicators and to contextualise the factors associated with victory or defeat (Sampaio and Janeira, 2003; Csataljay et al., 2009). Therefore, the situational variables have to be controlled when analysing futsal performance (Eccles et al., 2009; Gómez et al., 2013).

Thus, it is clear that the environment of performance, the characteristics of the tournament, and the culture of the sport or even the level of the competition in different countries should be considered (Eccles et al., 2009; Gómez et al., 2013). However, the comparison of the performance of elite futsal teams from different countries has been neglected in research. To our best knowledge, few attempts have been made to identify the physical, technical, and tactical performance that characterise elite futsal teams from different countries. In one study the authors compared the physical and technical indicators that differentiated elite futsal teams from Brazil, Spain, and Australia (Dogramaci et al., 2015). It revealed that Australian players spent significantly more time engaged in high-intensity activity than Spanish players (sprinting: 0.36 vs. 0.06%; running: 5.89 vs. 3.33%, *p* < 0.05). The Brazilian team showed the best passing accuracy and shot accuracy (10.7 ± 1.06 vs. 8.68 ± 0.81 vs. 5.31 ± 0.60, respectively, *p* < 0.01) and longer duration in ball possession than Spanish and Australian teams (40.0 ± 10.4% vs. 23.5 ± 2.73% vs. 30.9 ± 2.54%, respectively, *p* < 0.01). The Brazilian team showed the best passing accuracy and shot accuracy and longer duration in ball possession than Spanish and Australian teams. These results were used for comparison purposes according to the levels of the leagues of each country, and it was concluded that higher-performing teams (Brazilian and Spanish) are more accurate on the moves and on the passing and shooting actions than sub-elite-performing teams (Australian) (Dogramaci et al., 2015). In the same line of reasoning, Travassos et al. (2016) evaluated the flexibility/stability tendencies of the offensive play behaviours in six matches from the national futsal teams of Spain and Portugal through social network analysis. The authors aimed to observe the coordination trends emerging in the tactical behaviours of the players when facing different defensive formations of the opponent. The results showed similar network properties between teams when they compete against riskier defensive formations, but a greater level of adaptability for the unfolding of tactical variants of the Spanish team against conservative defences was observed in comparison with the Portuguese team. It was argued that such results help on the characterisation of the level of adaptability of the different countries’ teams to the futsal game variability and situational game environments (Travassos et al., 2016).

The available literature in futsal exploring team performance regarding task-related, situational, and contextual variables is limited (Moore et al., 2014). This is probably due to the lack of systematised information from futsal games and also due to the lack of researchers focusing on this sport in the past (Vicente-Vila and Lago-Peñas, 2016). The development of new studies in performance analysis of futsal that takes into account task-related, situational, and contextual variables is fundamental to understanding the game, identifying errors, and fundamentally perceiving its evolution (McGarry, 2009). Through these observations, coaches can identify the physical, technical, and tactical trends of the game that will allow a redefinition of game strategies to check and adapt the training routines to game demands (Niu et al., 2012; Morais et al., 2014).

Due to the unpredictable and complex nature of the sport of futsal (Vicente-Vila and Lago-Peñas, 2016), multivariate techniques for performance analysis are a useful tool when describing the normative profiles of the teams and their association with task-related, situational, and contextual variables. Accordingly, discriminant analysis has been suggested as a suitable statistical method for exploring and modelling such data in team sports (Sampaio and Janeira, 2003; Sampaio et al., 2006, 2018; Gómez et al., 2008; Ibáñez et al., 2009; Miloski et al., 2014) and examining differences between two or more groups with respect to several variables simultaneously (Klecka, 1980). For instance, previous research using this methodology in football revealed the following: the variables related to attacking play that best discriminate between winning and losing teams were goals scored, total shots, shots on goal, attacking moves, crosses, ball possession, match location, and quality of opposition. They emphasise the idea that coaches and players should be aware of these different attacking profiles in order to increase knowledge about game cognitive and motor solicitation and, therefore, to evaluate specificity in practise and game planning (Lago-Peñas et al., 2010, 2011; Lago-Peñas and Lago-Ballesteros, 2011; Castellano et al., 2012).

For the last two decades, Union of European Football Associations (UEFA) and Fédération Internationale de Football Association (FIFA) have promoted the implementation and development of futsal as a specific modality worldwide, although there are different levels of organisation and competitions at the amateur, semi-professional, and professional levels (Moore et al., 2014). The FIFA ranking recognises Brazil, Spain, Portugal, and Russia as the four best futsal national teams. Thus, their leagues, promoted by their national football federations, are presumed to represent the physical, technical, and tactical trends of the game (Göral, 2018). However, according to previous assumptions, task-related, situational, and contextual variables are fundamental for characterising such trends. Based on that, the aim of the present study was to identify the performance indicators that best discriminate the highest-ranked FIFA professional futsal leagues from three of the best four FIFA-ranked countries (Portuguese, Spanish, and Russian), as well as the variations that could be found when the condition of the winner, loser, or drawer is established. We expected to identify similarities between countries that help to characterise the trends of the game, but also to identify the characteristics that culturally define the national trends in the futsal game of Portuguese, Spanish, and Russian teams. Also, we expected to identify the variables that help to discriminate the winning, losing, or drawing teams in each country. Different results were expected to be observed among countries.

## Materials and Methods

### Ethical Approval

The Ethics Committee of Research and Development activities of UPM (Technical University of Madrid) was responsible for the evaluation of ethical aspects in order that data collection and processing used in this study will not affect fundamental rights. The ethics committee confirmed that the study respects the European data protection law (General Data Protection Regulation) regarding the public data processing of team sports. Once informed of the activity described in this manuscript, it was reviewed and approved by the Ethics Committee of Research and Development activities of UPM in order to be published.

Since the study involved analysis of not publicly available data, the requirement for informed consent was necessary. Astrofutsal^®^ and Instatscout^®^ acted as statistical suppliers of the elite futsal leagues to offer scouting services. Payment licences are necessary to access their datasets, and requests should be addressed through registration at http://www.astro-sport.com and https://www.instatscout.com. In order to avoid conflict of interests, steps were completed to buy a licence and to have access and use such data for research purposes. The informed consent of both companies was obtained in order that this study could use their statistical reports data in research, as well as in subsequent publications, in exchange for the source of the data repository being named.

### Data Sampling

A total of 56 matches corresponding to the playoffs from the 2017 to 2018 1st division of the Portuguese, Spanish, and Russian men’s elite futsal leagues were examined. The playoff league stage is played by the eight best-ranked teams during the regular season (played in a balanced schedule of 16 teams), and then the playoff includes the quarter-final, semi-final, and final rounds in a best-of-three series (five in the final round) in the Portuguese and Spanish leagues and in a best-of-five series in the Russian league with a home court advantage predetermined by the regular season results. One hundred and twelve cases were recorded in which the differences between the three European leagues were analysed with respect to variables linked to offensive game organisation and some situational variables.

### Data Processing

Regarding the data analysed in this study, the corresponding data on the Portuguese and Russian leagues were obtained through the Instat^®^ platform, and the data on the Spanish league were provided by the Astrofutsal^®^ platform. The data on the common variables in the match statistics of both platforms were observed, selected, and transferred to a unified matrix on an Excel spreadsheet. Astrofutsal^®^ (Méndez and Méndez, 2005) and Instatscout^®^ are common computer tools that provide data analysis of futsal competitions and are currently being used in sports research (Paz-Franco et al., 2014).

In the analysis of the reliability of the data, an expert observer from Astrofutsal^®^ analysed a match from the Portuguese league, crossing the records with those previously obtained in that match by the expert observer from Instatscout^®^. To carry out the interobserver reliability, the expert observer (12 years of experience in the notational analysis of futsal events with the use of the Astrofutsal^®^ tool) analysed the match between Sporting Club Portugal vs. Sport Lisboa e Benfica (1st match of the Playoff final series 2017–2018). The observer recorded the common events of the match for both teams, and the records were compared with those already established for that same game, using Cohen’s *Kappa* index (*k*) (Robinson and O’Donoghue, 2007). The results of the Kappa values from the events of both teams revealed very good agreement between both independent observers (*k* = 0.81 and *k* = 0.82) (Viera and Garrett, 2005).

### Data Notation

All the variables studied are defined in Table 1. The dependent variable was a categorical variable that determined the origin league of the best-ranked teams (eight best-ranked teams in each country, which were the ones that played the final playoff in the 2017–2018 season), being established in a polytomous contextual-dependent variable: Portugal, Spain, and Russia.

**Table 1:** Distribution of descriptive statistics from the studied variables.

Variables	Portugal *n* = 30				Spain *n* = 36				Russia *n* = 46			
	%	*n*	*X̄*	*SD*	%	*n*	*X̄*	*SD*	%	*n*	*X̄*	*SD*
Task related												
Efficiency												
Goals	–	88	2.9	1.8	–	102	2.8	1.3	–	140	3.0	1.9
Shots	–	1,161	38.7	14.8	–	1,243	34.5	8.8	–	2,127	46.2	8.9
Offensive organisation												
Counterattack	5.9	199	6.6	3.1	10.2	384	10.6	4.5	6.0	406	8.8	3.7
Positional attack	71.5	2,402	80.0	15.2	57.4	2,170	60.2	12.5	70.5	4,750	103.2	12.7
Set pieces	17.5	588	19.6	8.7	27.7	1,051	29.1	10.5	18.4	1,241	26.9	6.5
5vs4+Gk	5.1	170	5.6	6.4	4.7	178	4.9	6.6	5.1	339	7.3	9.2

Situational												
1st goal												
Home team	53.3	16	–	–	55.6	20	–	–	65.2	30	–	–
Away team	46.7	14	–	–	44.4	16	–	–	34.8	16	–	–
Match outcome												
Winner	50.0	15	–	–	44.4	16	–	–	47.8	22	–	–
Loser	50.0	15	–	–	44.4	16	–	–	47.8	22	–	–
Drawer	–	–	–	–	11.1	4	–	–	4.3	2	–	–
Match type												
Balanced	73.3	22	–	–	77.8	28	–	–	56.5	26	–	–
Unbalanced	26.7	8	–	–	22.2	8	–	–	43.5	20	–	–

The independent or discriminating variables were presented as task-related variables and situational variables. The task-related variables (continuous) included variables related with efficiency of teams: (i) goals (number of goals scored by each team at the end of the match); (ii) shots (number of shots made by each team, without taking into account their final effects, although as the goal is almost always preceded by a shot, has been considered that the goals scored are included in the shots count); and variables related with the attacking teams’ organisation; (iii) positional attack (number of times the team uses this type to end the attack), considered when the attacking players are ordered according to the ball and try to progress to the opposing goal with offensive means to unbalance a positioned opponent defence in front of the ball; (iv) counterattack (number of counterattacks carried out) considered at the attempt to exploit the free spaces caused by the absence of the adversary collective fallback, where speed and depth predominate in the game after ball recovery; (v) set pieces (total sum of actions to set pieces including throw-ins, corners, penalty free kick from the second penalty mark at 10 m, and free kick with opposition); and (vi) 5vs4+GK (number of times the team uses this strategy in the match). The situational variables (categorical) included (vii) the 1st goal scored in the match (that differentiates the teams that scores a goal before their opponent playing at home or away); (viii) match outcome (that differentiates winners, losers, and drawers, once the periods of play time have ended); and (ix) match type [that discriminates the balance between teams over the game (balanced game: with differences of up to two goals, and unbalanced games: more than a two-goal difference)].

### Statistical Analysis

Firstly, the relationships of the dependent variable (teams from different leagues) with each of the independent categorical variables were analysed, taken one by one, using crosstabs. For each relationship, the Pearson chi-square test and its symmetric measurements were used in order to see significant effects between the teams of the different leagues and situational variables. The effect sizes (ESs) were calculated using Cramer’s *V* test, and their interpretation was based on the following criteria: 0.10 = small effect, 0.30 = medium effect, and 0.50 = large effect (Volker, 2006).

Secondly, a discriminant analysis was used on the performance of the teams from the three different leagues considered to create a function that classifies the teams from each league as accurately as possible. The fact that the teams come from different leagues was used as a dependent variable, while the variables in which differences were assumed were used as independent variables. In each of the three groups (European futsal leagues), two discriminant functions were obtained and interpreted based on the examination of structure coefficients (SCs) greater than |0.30| (Tabachnick and Fidell, 2007). The current study basically consists of two discriminant analyses: First, a previous analysis tried to find out how the Portuguese, Spanish, and Russian teams differ with respect to their attacking game organisation and its effectiveness. After, a subsequent analysis tried to ascertain if there was any variable that could discriminate the teams when the condition of a winner or a non-winner was included.

The statistical specifications of the model included (i) an analysis of variance (ANOVA) test with *F* statistics that contrasts the equality of means hypothesis among the groups in each independent variable (it served as a preliminary test to detect if the groups differ significantly (*P <* 0.05) in the classification variables); (ii) the eigenvalues show the canonical correlation, whose value (between 0 and 1) indicates to what extent the discriminant variables make it possible to differentiate among the three groups; (iii) *Wilks’ Lambda*, which expresses the total variability proportion not due to the differences among the groups, making it possible to contrast the null hypothesis that the means of the groups are equal and establish the discriminant functions of the model (1 and 2) associated with a critical level of significance (*P* < 0.05); (iv) group centroids show the location of the teams in each of the two discriminant functions, making it possible to see if they are located, on average, in the positive or negative scores of the function (the 1st function explains the maximum possible differences among groups, and the second function explains the maximum of the unexplained differences until reaching 100%); (v) the standardised coefficients determine the net contribution of each variable when predicting the group to which the teams belong; (vi) the SCs determine the correlation of the variables with the discriminant functions (1 and 2), those of the 1st function being the ones with the greatest discriminative capacity (the larger the magnitude of the coefficients, the greater the contribution of that variable to the discriminant function, showing the ones that contribute most to discriminating from the value ≥|0.30|); (vii) the classification statistics make it possible to assess the predictive capacity of the estimated model [previous or *a priori* probabilities indicate that the same relative importance has been given to all leagues (0.333) regardless of the sample size]; and (viii) the classification results indicate the % of cases correctly classified in relation to the 33% expected in a completely randomised classification. Statistical analyses were performed using IBM SPSS for Windows statistics, version 22.0 (IBM Corp., Armonk, NY, United States).

## Results

The distributions of relative frequencies from the studied variables are shown in Table 1. The results revealed (i) a goal average per match very similar among leagues (Portugal 2.9, Spain 2.8, and Russia 3.0); (ii) the highest frequency of offensive organisation is positional attack in all the leagues (Portugal 71.5%, Spain 57.4%, and Russia 70.5%); (iii) usually, the home team is the first to score in all the leagues (Portugal 53.3%, Spain 55.6%, and Russia 65.2%); and (iv) the majority of matches were characterised as balanced matches (Portugal 73.3%, Spain 77.8%, and Russia 56.5%).

Results from the crosstabs showed that the first goal scored by the home or away team did not significantly influence the team performance in the different leagues [Table 2: *p*-value = 0.520; ES (*V)* = 0.10], nor were significant differences found in the team performance in the different leagues in the case of the match type [Table 3: *p*-value = 0.093; ES (*V*) = 0.20].

**Table 2:** Dependence relationship between the 1st goal scored and the European leagues.

1st goal scored		Portugal	Spain	Russia	Total
Home team	Count	16	20	30	66
	% within league	53.3%	55.6%	65.2%	58.9%
	Corrected residue	−0.7	−0.5	1.1	–
Away team	Count	14	16	16	46
	% within league	46.7%	44.4%	34.8%	41.1%
	Corrected residue	0.7	0.5	−1.1	–
Total	Count	30	36	46	112
	% within league	100.0%	100.0%	100.0%	100.0%

**Chi-square tests and symmetric measurements**					

***χ*^2^**	***df***	**Sig**	**Mef**	***V***

1.309	2	0.520	12.32	0.10

*^∗^P < 0.05; Sig, significance; df, degrees of freedom; Mef, minimum expected frequency; Cramer’s V test, effect size.*

**Table 3:** Dependence relationship between the match type and the European leagues.

Match type		Portugal	Spain	Russia	Total
Balanced	Count	22	28	26	76
	% within league	73.3%	78.8%	56.5%	67.9%
	Corrected residue	0.8	1.5	−2.1	–
Unbalanced	Count	8	8	20	36
	% within league	26.7%	22.2%	43.5%	32.1%
	Corrected residue	−0.8	−1.5	2.1	–
Total	Count	30	36	46	112
	% within league	100.0%	100.0%	100.0%	100.0%

**Chi-square tests and symmetric measurements**					

***χ*^2^**	***df***	**Sig**	**Mef**		***V***

4.447	2	0.093	9.64		0.20

*^∗^P < 0.05; Sig, significance; df, degrees of freedom; Mef, minimum expected frequency; Cramer’s V test, effect size.*

Additionally, no significant relationship was observed between the winner–nonwinner condition of the teams from the different leagues and the team that scored the first goal of the match {Table 4: Portugal [*p*-value = 1.0; ES(*V*) = 0.00]; Spain [*p*-value = 0.060; ES(*V*) = 039]; and Russia [*p*-value = 0.141; ES(*V*) = 0.29]}.

**Table 4:** Dependence relationship between the winner–nonwinner condition and the 1st goal scored.

Match outcome		1st goal scored		Total	
		Home team	Away team		
Winner	Count	33	20	53	
	% within 1st goal	50.0%	43.5%	47.3%	
	Corrected residue	0.7	−0.7	–	
Loser	Count	33	20	53	
	% within 1st goal	50.0%	43.5%	47.3%	
	Corrected residue	0.7	−0.7	–	
Drawer	Count	0	6	6	
	% within 1st goal	0.0%	13.0%	5.4%	
	Corrected residue	0.0	3.0	–	
Total	Count	66	46	112	
	% within 1st goal	100.0%	100.0%	100.0%	

**Chi-square tests and symmetric measurements**					

**Country**	***χ*^2^**	***df***	**Sig**	**Mef**	***V***

Portugal	0.000	1	1.0	7	0.00
Spain	5.625	2	0.060	1.78	0.39
Russia	3.920	2	0.141	0.70	0.29

*^∗^P < 0.05; Sig, significance; df, degrees of freedom; Mef, minimum expected frequency; Cramer’s V test, effect size.*

**FIGURE 1:**
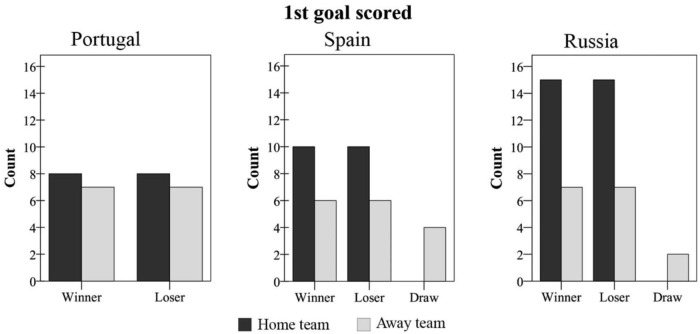
Influence of the first goal scored in the winner–nonwinner condition in the Portuguese **(left)**, Spanish **(middle)**, and Russian **(right)** leagues.

The odds of scoring the first goal of the match were better in the home than in the away teams in all leagues. However, these differences do not make it possible to establish the final condition of the winner or loser, and therefore, no significant relationship was observed between both variables (Figure 1).

Secondly, the previous discriminant analysis revealed differences in the attacking game organisation of the Portuguese, Spanish, and Russian teams. The ANOVA revealed that teams from the different leagues differ significantly in four of the selected classification variables: shots, positional attack, counterattack, and set pieces (Table 5).

**Table 5:** ANOVA preliminary test with *F* statistics that contrasts the equality of means hypothesis among the groups in each independent variable.

Variables	Wilks’ Lambda	*F*	df1	df2	Sig.
Goals	0.997	0.145	2	109	0.865
Shots	0.816	12.324	2	109	0.000^∗^
Positional Attack	0.341	105.266	2	109	0.000^∗^
Counterattack	0.862	8.753	2	109	0.000^∗^
Set Pieces	0.831	11.059	2	109	0.000^∗^
5vs4+Gk	0.981	1.050	2	109	0.353

*^∗^p < 0.01. ^∗∗^p < 0.05.*

The summary of discriminant functions showed that the first function explained almost 90% of the data variability, while the second function 10.4%. Both functions make it possible to differentiate significantly among the teams of the three leagues. The first function fundamentally differentiates the Spanish teams from the Portuguese and Russian teams. The standardised coefficients showed that the first function discriminates between teams that played with more positional attacks and fewer counterattacks (Russian and Portuguese), and the second function between teams with more set pieces and fewer shots (Russian). The SCs corroborate the high correlation of the positional attack with the first function, and the set pieces and counterattacks with the second function (Table 6).

**Table 6:** Summary of discriminant functions.

Variables	Eigenvalues and *Wilks’ Lambda*		Group centroids		Standardised coefficients		SCs	
	Function		Function		Function		Function	
	1	2	1	2	1	2	1	2
Portugal	–	–	0.438	– 1.061	–	–	–	–
Spain	–	–	–2.654	0.269	–	–	–	–
Russia	–	–	1.791	.481	–	–	–	–

Positional Attack	–	–	–	–	1.390	0.627	**0.698**^∗†^	0.511
Goals	–	–	–	–	–0.207	0.062	0.026^∗^	0.018
Set Pieces	–	–	–	–	–0.340	1.262	−0.088	0.**634**^∗†^
Counterattack	–	–	–	–	–0.608	0.495	−0.129	0.**478**^∗†^
Shots	–	–	–	–	–0.271	−1.218	0.231	0.248^∗^
5vs4+Gk	–	–	–	–	0.164	0.479	0.066	0.084^∗^

Eigenvalue	3.733	0.432	–	–	–	–	–	–
% of Variance	89.6%	10.4%	–	–	–	–	–	–
Canonical correlation	0.888	0.549	–	–	–	–	–	–
Wilks’ Lambda	0.148	0.698	–	–	–	–		
Chi-Square	201.870	37.867	–	–	–	–	–	–
*Df*	16	7	–	–	–	–	–	–
Significance	0.000	0.000	–	–	–	–	–	–

*^†^SC discriminant value ≥|0.30|. ^∗^The highest absolute correlation between each variable and any discriminant function.*

**FIGURE 2:**
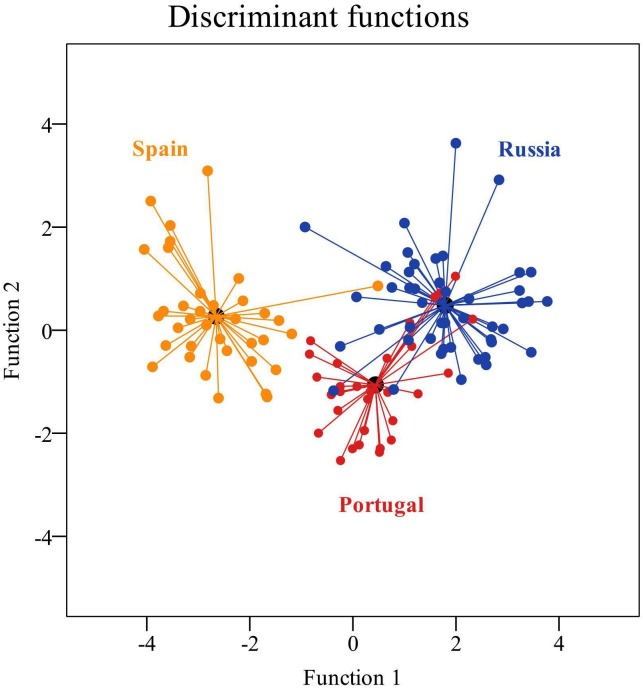
Territorial map of the discriminant functions for the Portuguese, Spanish, and Russian leagues.

The explanatory capacity of the model indicates that 88.4% of the cases were correctly classified, with the highest percentage of correct classifications for the Spanish league teams, which are not to be confused with the Russian and Portuguese teams, which are more similar to each other (Figure 2).

The subsequent discriminant analysis compared the teams from the different leagues, trying to differentiate them by their status as winner, loser, or drawer. The results showed that the 5vs4+GK strategy and the goals are the variables that most contribute to discriminating the teams with respect to the condition of winner–nonwinner (Table 7).

**Table 7:** ANOVA preliminary test with *F* statistics that contrasts the equality of means hypothesis among the groups in each independent variable.

League	Variables	Wilks’ Lambda	*F*	df1	df2	Sig.
Portugal	Goals	0.549	22.967	1	28	0.000^∗^
	Shots	0.661	14.345	1	28	0.001^∗^
	Positional attack	0.988	0.350	1	28	0.559
	Counterattack	0.959	1.199	1	28	0.283
	Set Pieces	0.747	9.503	1	28	0.005^∗^
	5vs4+Gk	0.300	65.401	1	28	0.000^∗^
Spain	Goals	0.442	20.791	2	33	0.000^∗^
	Shots	0.898	1.867	2	33	0.171
	Positional attack	0.782	4.608	2	33	0.017^∗∗^
	Counterattack	0.962	0.644	2	33	0.531
	Set Pieces	0.856	2.774	2	33	0.077
	5vs4+Gk	0.374	27.622	2	33	0.000^∗^
Russia	Goals	0.560	16.926	2	43	0.000^∗^
	Shots	0.964	0.797	2	43	0.457
	Positional attack	0.813	4.959	2	43	0.012^∗∗^
	Counterattack	0.950	1.136	2	43	0.331
	Set Pieces	0.984	0.343	2	43	0.712
	5vs4+Gk	0.422	29.431	2	43	0.000^∗^

*^∗^p < 0.01. ^∗∗^p < 0.05.*

The summary of the discriminant functions (Table 8) showed that for Portugal, only the first discriminant function explains 100% of the variability, while Spain and Russia required both discriminant functions. Each and every one of the functions, with the exception of the second Russian discriminating function, shows significant differences between the winning and non-winning teams. In all leagues, the first function classifies winning teams from non-winning teams. The standardised coefficients showed that for Portugal and Spain, the first function essentially discriminates between the teams with most shots and playing more with 5vs4+GK and less with set pieces and positional attack (losers). Similarly, in Russia, the first function discriminates between the teams playing more with 5vs4+GK but less with counterattacks (losers). The SCs showed in all leagues the highest absolute correlation of the 5vs4+GK strategy and goals with the main discriminant function and were also the variables that most contribute to discriminating between winning and non-winning teams. In addition, the Spanish league established the highest correlation of positional attacks and set pieces with the second discriminant function (Table 8).

**Table 8:** Summary of discriminant functions.

League	Variables	Eigenvalues and *Wilks’ Lambda*		Group centroids		Standardised coefficients		SCs	
		Function		Function		Function		Function	
		1	2	1	2	1	2	1	2
Portugal	Winner	–	–	1.918	–	–	–	–	–
	Loser	–	–	−1.918	–	–	–	–	–
	Drawer	–	–	–	–	–	–	–	–
	5vs4+Gk	–	–	–	–	−0.617	–	−**0.770**^∗†^	–
	Goals	–	–	–	–	0.350	–	**0.456**^∗†^	–
	Shots	–	–	–	–	−2.150	–	**0.361**^∗†^	–
	Set Pieces	–	–	–	–	0.810	–	0.293	–
	Counterattack	–	–	–	–	0.366	–	0.104	–
	Positional attack	–	–	–	–	0.608	–	0.056	–
	Eigenvalue	3.941	–	–	–	–	–	–	–
	% of Variance	100%	–	–	–	–	–	–	–
	Canonical correlat.	0.893	–	–	–	–	–	–	–
	Wilks’ Lambda	0.202	–	–	–	–	–	–	–
	Chi-Square	38.343	–	–	–	–	–	–	–
	*Df*	8	–	–	–	–	–	–	–
	Significance	0.000	–	–	–	–	–	–	–
Spain	Winner	–	–	2.258	0.283	–	–	–	–
	Loser	–	–	−2.141	0.462	–	–	–	–
	Drawer	–	–	−0.469	−2.980	–	–	–	–
	5vs4+GK	–	–	–	–	−0.439	0.193	−**0.566**^∗†^	0.367
	Goals	–	–	–	–	0.816	0.330	**0.504**^∗†^	0.225
	Counterattack	–	–	–	–	0.549	0.644	0.086^∗^	−0.056
	Positional attack	–	–	–	–	1.277	−0.545	0.048	−**0.469**^∗†^
	Set Pieces	–	–	–	–	1.480	−0.419	−0.037	−**0.364**^∗†^
	Shots	–	–	–	–	−3.530	−1.469	0.071	−0.271^∗^
	Eigenvalue	4.720	1.219	–	–	–	–	–	–
	% of Variance	79.5%	20.5%	–	–	–	–	–	–
	Canonical correlat.	0.908	0.741	–	–	–	–	–	–
	Wilks’ Lambda	0.79	0.451	–	–	–	–	–	–
	Chi-Square	74.958	23.508	–	–	–	–	–	–
	*Df*	16	7	–	–	–	–	–	–
	Significance	0.000	0.001	–	–	–	–	–	–
Russia	Winner	–	–	1.619	0.102	–	–	–	–
	Loser	–	–	−1.631	0.086	–	–	–	–
	Drawer	–	–	0.134	−2.062	–	–	–	–
	5vs4+Gk	–	–	–	–	−0.671	0.583	−**0.708**^∗†^	0.258
	Goals	–	–	–	–	0.551	−0.036	**0.534**^∗†^	−0.270
	Counterattack	–	–	–	–	0.186	0.532	0.139^∗^	0.052
	Positional attack	–	–	–	–	−0.098	−0.150	−0.188	−**0.808**^∗†^
	Shots	–	–	–	–	−0.583	−2.378	0.073	−**0.331**^∗†^
	Set Pieces	–	–	–	–	−0.076	0.554	0.031	−0.254^∗^
	Eigenvalue	2.703	0.207	–	–	–	–	–	–
	% of Variance	92.9%	7.1%	–	–	–	–	–	–
	Canonical correlat.	0.854	0.414	–	–	–	–	–	–
	Wilks’ Lambda	0.224	0.829	–	–	–	–	–	–
	Chi-Square	59.140	7.425	–	–	–	–	–	–
	*Df*	16	7	–	–	–	–	–	–
	Significance	0.000	0.386	–	–	–	–	–	–

*^†^SC discriminant value ≥|0.30|. ^∗^The highest absolute correlation between each variable and any discriminant function.*

**FIGURE 3:**
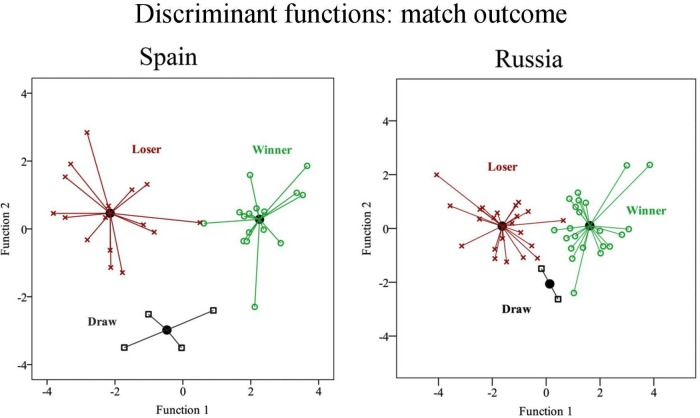
Territorial map of the discriminant functions according to match outcome for the Spanish and Russian leagues.

The explanatory capacity of the model indicates that Portugal correctly classifies 100% of cases, Spain 97.2%, and Russia 89.1%. Dispersion diagrams showed that it is easier to differentiate winning from non-winning teams in Spain than in Russia (Figure 3).

## Discussion

The aim of this study was to identify the performance indicators that best discriminate the best-ranked FIFA professional futsal leagues from three of the best four FIFA-ranking countries (Portugal, Spain, and Russia), as well as the variations that could be found when the condition of the winner, loser, or drawer is established. According to our expectations, despite considering three of the best leagues, it was possible to identify characteristics that culturally define the national trends in futsal of Portuguese, Spanish, and Russian teams. The crosstab results do not establish dependency between the leagues and any of the situational variables. The discriminant analysis results indicated that the Portuguese and Russian teams used more positional attacks and less counterattacks and set pieces in relation to the Spanish teams. However, the same was not observed in the analysis of winners, losers, or drawers among leagues. Generally, the results revealed that the 5vs4+GK strategy and the number of goals obtained were the most powerful match variables for discriminating losing and winning teams in the three leagues.

The analysis of the situational variables did not reveal differences between the three leagues analysed. That is, for the Portuguese, Spanish, and Russian leagues, the home teams revealed a greater frequency in scoring the first goal than the away teams, and balanced matches were recorded more often than unbalanced matches. In fact, the differences observed do not reflect a trend that makes it possible to establish a relationship between first goal, match type, and match outcome for all the leagues considered. However, these results do not agree with previous investigations, where the match location had an influence and the effect of scoring the first goal showed a significant relationship with the victory in the final result of the match in teams from the Spanish futsal league (Sampedro and Prieto, 2012). Further research is required considering teams of different levels and from different moments of game competition (regular phase or play-off).

The results of the discriminant analysis indicated that the Portuguese and Russian teams used more positional attacks and less counterattacks and set pieces than the Spanish teams. The contribution of the so-called organised game or positional attack to the end of the offensive phase in elite futsal matches is well documented (Alvarez et al., 2004; Silva et al., 2004; Soares-Leite, 2012; Ribeiro et al., 2014; Sarmento et al., 2016). The results revealed that positional attack is the type of attack most used by the teams and, together with counterattack and the set pieces, provides the greatest number of goals (Alvarez et al., 2004; Silva et al., 2004; Soares-Leite, 2012; Ribeiro et al., 2014; Sarmento et al., 2016). Trying to establish similarities with previous results from Travassos et al. (2016), which observe that the Spanish national futsal team had higher variability in the positional attack than the Portuguese national team, our results also seem to reveal that Spanish teams used positional attacks, counterattacks, and set pieces with more variability according to the contextual match dynamics than Portuguese and Russian teams. In agreement, the findings may be suggesting that Spanish teams employ a greater variety of tactical resources, promoting higher adaptability in collective behaviour.

The variations found in the discriminant analysis, when the winning, losing, or drawing condition is established, showed that the 5vs4+GK strategy was the variable that reaches the greatest magnitude and weighting in the SCs to discriminate the losing teams in all the leagues. As was expected, goals scored discriminate winning teams, in accordance with a previous study by Lago-Peñas and Lago-Ballesteros (2011). From the findings observed, the Portuguese, Spanish, and Russian losing teams used the 5vs4+GK strategy much more than the winning teams. It is a conventional strategy used by futsal coaches when losing the game in order to promote numerical superiority, with the aim of recovering the balance on the scoreboard (Travassos et al., 2011). However, usually, this type of strategy cannot help teams to change the match outcome and possibly means finishing the game with more goals received than scored. Recent research reinforces the idea that teams that use the 5vs4+GK do not alter the match outcome with respect to match status. Although it is a risk strategy that can provide superiority and profitability, the likelihood of changing the final match status is scarce, and the teams that were losing and used the 5vs4+GK game strategy lost the game in 93% of cases and received more goals compared to those scored (Méndez, 2017). Thus, according to such results, it cannot be considered an effective strategy for winning matches (Göral, 2018).

Additionally, previous research has considered that the 5vs4+GK game strategy does not make it possible to achieve the same number of goals when compared to the counterattack, positional attack, and set pieces (Silva et al., 2004; Botelho and Coppi, 2010; Marchi et al., 2010; Fukuda and Santana, 2012; Poffo and Lima, 2012; Soares-Leite, 2012). In agreement, other authors found a similar percentage of goals scored and received with the 5vs4+GK strategy (Alvarez et al., 2004; Barbosa, 2011).

In the end, losing teams are well discriminated in all leagues through the use of the 5vs4+GK game strategy. That is, this game strategy was almost always used by the teams that were losing the game and did not allow them to change the final game status. Previous research revealed that this game strategy is more profitable when it is used during the game when the attacking team is winning or drawing than at the end of the game when the attacking team is losing (Méndez, 2017). Thus, coaches should use this information to prepare the training sessions and the use of different game strategies according to variations in task-related and situational game variables (Ganef et al., 2009; Newton-Ribeiro, 2011).

The discriminatory power of the attacking game profiles of teams from elite futsal leagues made it possible to understand that the Spanish teams have a more balanced profile than the Portuguese and Russian teams when facing the end of the offensive phase. This means that Spanish players are better able to adapt to the variability of the competition, allowing them greater heterogeneity not only to take the initiative but also to wait and see what the other team does in attacking situations and take the most suitable action depending on the trend of the game. These results suggest that regarding winning, drawing, and losing, the national futsal teams may be discriminated from one another on the basis of variables such as ball possession and the effectiveness of their attacking play.

The present study has the limitation of only considering games from the play-off of the three elite futsal leagues analysed. Consequently, the characterisation of each futsal league may be biassed due to only considering the best eight ranked teams playing play-off games. Further research should consider the analysis of the entire leagues to improve the comparison. Also, further research should be developed considering other task-related variables and situational variables that help to improve the tactical characterisation of the teams’ behaviour. For instance, the aspects that discriminate the teams from the different leagues between winners and non-winners should be expanded to recognise the importance of the match type, so it would be interesting to create subcategories that indicate if that final condition was achieved within a match balanced (up to 2 goals) or unbalanced (above 2 goals difference). It will help to not only to describe but also to explain the main differences obtained (Travassos et al., 2013). In the same way, it would be interesting to note which tactical systems were used in organised attack (1:3:1 or 1:4:0), whether the counterattack was in superiority (1vs0+GK, 2vs1+GK)…, or in numerical equality (1vs1+GK or 2vs2+GK…), which were the set pieces that resulted in more goals or the offensive or defensive spatial–temporal relations that characterise each pattern of play (space covered, distances between players, zones of the court used…).

## Author Contributions

CM and BT contributed to the conception and design of the study. CM, JS, and BG collected the data and performed the statistical analysis. CM, BT, and JR wrote the manuscript. JS, JR, and BT revised and finalised the manuscript. CM organised the database. All the authors contributed to the manuscript revision and read and approved the submitted version.

## Conflict of Interest Statement

The authors declare that the research was conducted in the absence of any commercial or financial relationships that could be construed as a potential conflict of interest.
